# A Rare Case of Chicken-Bone-Induced Esophageal Perforation Leading to Cervical Abscess and Esophagocutaneous Fistula

**DOI:** 10.7759/cureus.105525

**Published:** 2026-03-19

**Authors:** Garrett Teskey, Oneka Daniels, Zoilo Augusto Placeres Leon, Mazer Ally

**Affiliations:** 1 Internal Medicine, Scripps Mercy Hospital, San Diego, USA; 2 Gastroenterology, Georgetown Public Hospital Corporation, Georgetown, GUY; 3 Surgery, Georgetown Public Hospital Corporation, Georgetown, GUY; 4 Gastroenterology and Hepatology, Scripps Clinic, San Diego, USA

**Keywords:** cervical abscess, esophageal perforation, esophagocutaneous fistula, esophagus, foreign body

## Abstract

Foreign body (FB) ingestion is a frequent clinical occurrence, but most objects pass spontaneously without complication. Sharp foreign bodies, particularly fish or chicken bones, significantly increase the risk of esophageal perforation, a rare but potentially fatal event. This report describes a 45-year-old woman who presented repeatedly with sore throat, dysphagia, low-grade fevers, weight loss, and a palpable cervical mass after ingesting a chicken bone. Initial evaluations were unrevealing. On gastroenterology referral, her exam revealed left cervical swelling, endoscopy demonstrated purulent drainage from the proximal esophagus, and bedside ultrasound uncovered a complex peri-esophageal fluid collection with air pockets and a suspected retained FB. Aspiration produced purulent fluid concerning an esophagocutaneous fistula. Surgical exploration revealed a deep cervical abscess, and a chicken bone fragment was removed. The esophageal defect was repaired with muscle flap interposition, and a gastrostomy tube was placed for feeding. The patient recovered well, with no postoperative leak on barium swallow.

This case highlights the diagnostic challenges of cervical esophageal perforation, particularly when early symptoms are nonspecific and initial imaging is negative. A bedside ultrasound proved valuable in identifying the retained FB and guiding timely intervention. Although delayed diagnosis increases risk, coordinated multidisciplinary management can still achieve excellent outcomes, as shown by this.

## Introduction

Foreign body (FB) ingestion is a common clinical problem, with most objects passing spontaneously, but sharp objects can result in severe complications, especially when lodged in the esophagus [1]. Sharp FBs such as fish or chicken bones increase the risk of mucosal injury and esophageal perforation, with mortality rates approaching 20% when diagnosis is delayed [2-5]. Complications may include abscesses, fistulas, mediastinitis, and vascular injury [6,7]. 

Cervical esophageal perforation can lead to deep neck space infections, including cervical abscesses, which pose a significant threat due to the region’s proximity to critical vascular and airway structures. If untreated, these infections can extend into the mediastinum or cause airway compromise. One severe sequela is the development of an esophagocutaneous fistula, a pathological tract between the esophagus and skin, often resulting from prolonged inflammation or infection secondary to delayed diagnosis. Such fistulas are rare but can lead to persistent drainage, nutritional compromise, and require surgical intervention for resolution. Early recognition, imaging, and multidisciplinary management are essential for favorable outcomes [2,8]. We present a rare case of delayed proximal esophageal perforation by a chicken bone, which progressed to cervical abscess and esophagocutaneous fistula requiring surgical repair.

## Case presentation

**Figure 1:**
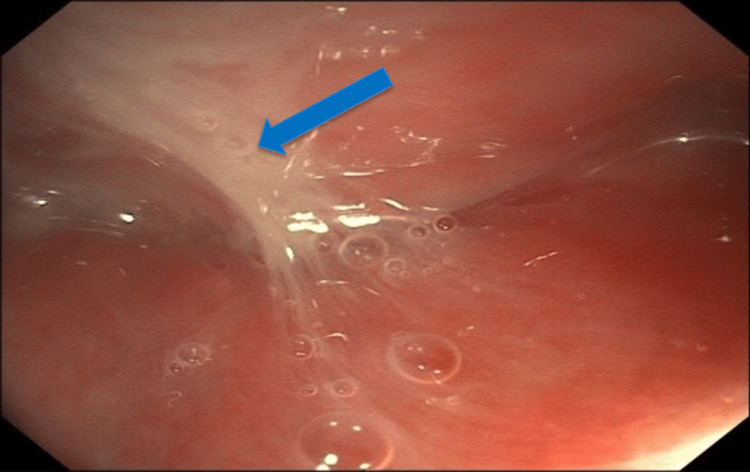
Upper endoscopy showing purulent drainage in the esophagus Endoscopic view of the proximal esophagus demonstrating purulent discharge. The blue arrow highlights the area of white, purulent material draining into the esophageal lumen.

A 45-year-old woman with no significant prior medical history presented to the emergency department multiple times over the course of several days, reporting approximately 21 days of progressive dysphagia and odynophagia, reduced oral intake resulting in weight loss, low-grade fevers, and a palpable cervical neck mass. She reported recent ingestion of a chicken bone, but the initial physical examinations were unremarkable, and she was repeatedly discharged with conservative management. Due to persistent symptoms, the patient was referred to gastroenterology for evaluation. Examination revealed swelling and tenderness in the left cervical region. Upper endoscopy showed purulent secretions draining from the proximal esophagus into the stomach (Figure 1). A fistulous opening was identified at approximately 25 cm from the incisors, from which purulent material was seen draining. The remainder of the esophagus appeared grossly normal, with no additional ulcers or mucosal abnormalities noted. Gastric biopsies were obtained during endoscopy as part of a routine evaluation for upper GI symptoms and revealed chronic gastritis without evidence of *Helicobacter pylori* infection.

**Figure 2:**
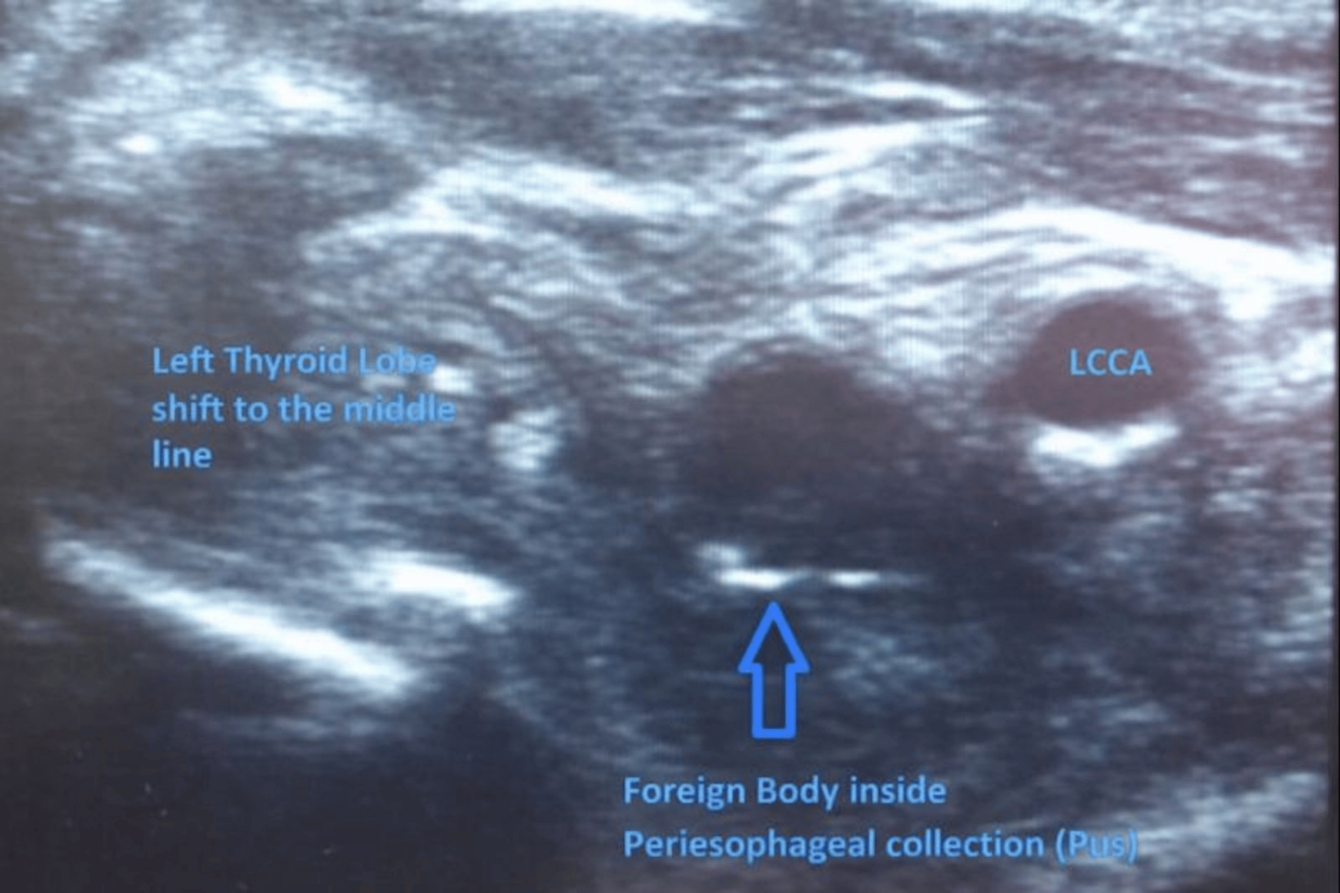
Point-of-care ultrasound of the left cervical region demonstrating a complex peri-esophageal fluid collection with internal air pockets and a linear hyperechoic structure consistent with a retained foreign body external to the esophageal wall (arrow) LCCA, left common carotid artery

A point-of-care ultrasound (POCUS) revealed a complex peri-esophageal fluid collection with air pockets and a suspected FB external to the esophageal wall (Figure 2). Aspiration of the collection via a 16-gauge intracath yielded approximately 10 mL of purulent fluid, which raised concern for an esophagocutaneous fistula.

**Figure 3:**
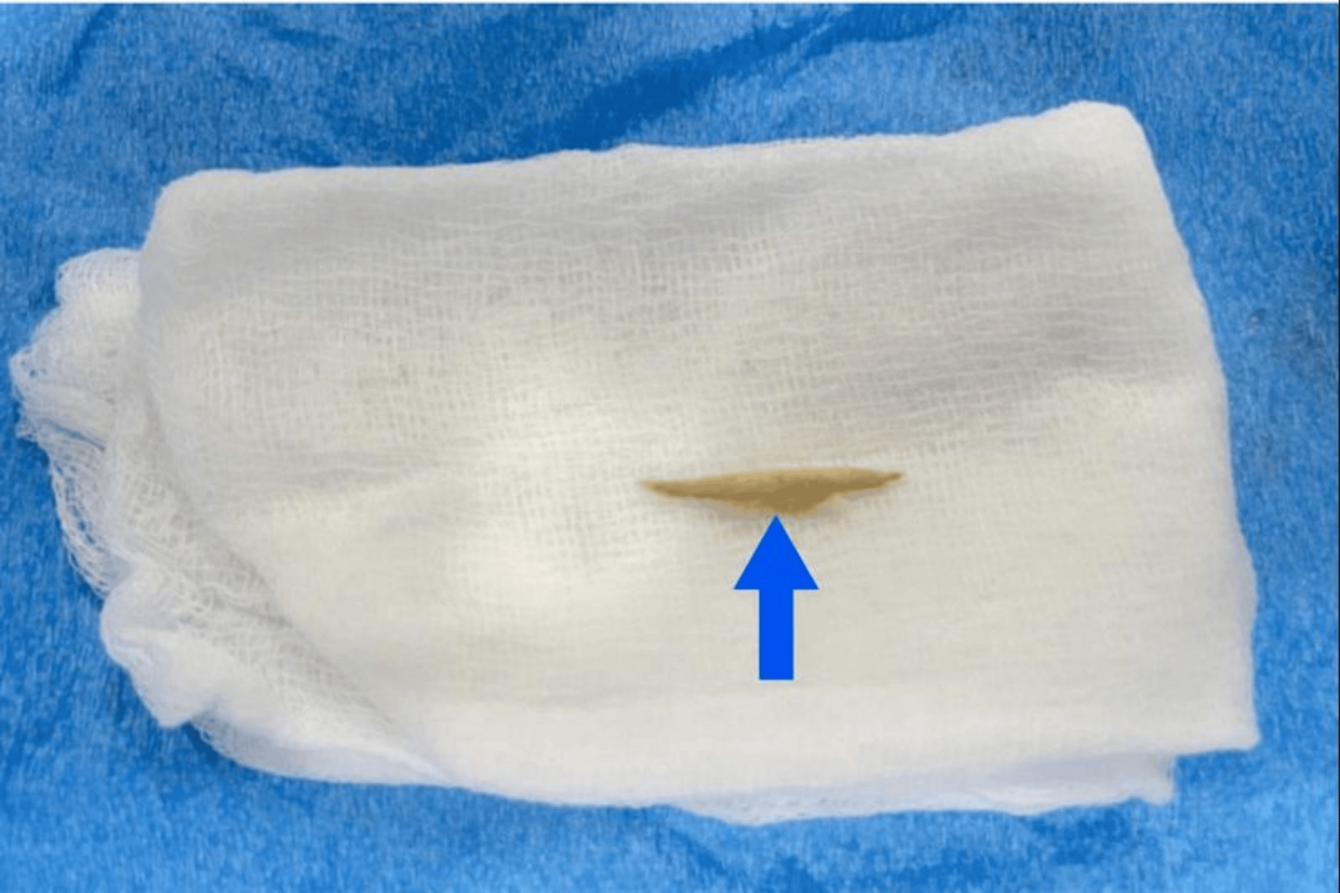
Surgically extracted chicken bone fragment (blue arrow) responsible for proximal esophageal perforation and cervical abscess

The patient underwent a left neck exploration, which revealed a deep neck abscess. Surgical debridement was performed, and the chicken bone fragment was retrieved (Figure 3). The esophagus was repaired with a muscle flap interposition, and a gastrostomy tube was placed to provide longer-term enteral nutrition, as the esophageal perforation and fistula precluded oral intake. A nasogastric tube was placed temporarily for gastric decompression in the acute postoperative period and was removed the following day once decompression was no longer required. Postoperatively, the patient recovered well, tolerated gastrostomy feeds, and a barium swallow showed no leak. She was discharged with outpatient follow-up for eventual gastrostomy removal.

## Discussion

Esophageal perforation due to chicken bone ingestion poses a significant diagnostic challenge, especially when initial symptoms are nonspecific and initial imaging is inconclusive. Sharp objects like bones are more likely to cause perforation, particularly in the cervical esophagus, which is anatomically vulnerable due to its proximity to vital structures and limited tissue coverage [9-12].

In this case, delayed diagnosis led to severe complications, including abscess formation and fistula development. The use of POCUS was instrumental in identifying a deep neck abscess and suspicion of an FB. While plain radiographs can miss esophageal perforations, ultrasound and CT are more sensitive for identifying soft tissue changes and collections [8,13,14].

Management depends on timing, location, and extent of injury. Surgical intervention remains the cornerstone of treatment for complicated esophageal perforation, which may include primary repair, drainage, and supportive measures such as a feeding gastrostomy [2,5,6,15]. The literature suggests early surgical repair within 24 hours for better outcomes [5,16]. However, this case demonstrates that delayed intervention can still lead to a successful recovery.

## Conclusions

Clinicians should maintain a high index of suspicion for esophageal perforation in patients with persistent symptoms following ingestion of sharp FBs, even when early evaluations are benign. Early imaging, endoscopic evaluation, and timely surgical management are crucial to prevent severe complications. POCUS may serve as a valuable initial tool in resource-limited settings or as an adjunct to clinical evaluation. With appropriate multidisciplinary intervention, even delayed presentations can achieve excellent outcomes, as demonstrated by this patient’s clinical stabilization, recovery, and plans to resume oral intake following gastrostomy support.
